# An in vitro ULV olfactory bioassay method for testing the repellent activity of essential oils against moths

**DOI:** 10.1016/j.mex.2018.03.012

**Published:** 2018-04-20

**Authors:** Petros T. Damos

**Affiliations:** Laboratory of Applied Zoology and Parasitology, Department of Crop Production (Field Crops and Ecology, Horticulture and Viticulture and Plant Protection), Faculty of Agriculture, Forestry and Natural Environment, Aristotle University of Thessaloniki, University Campus, 541 24, Thessaloniki, Greece

**Keywords:** Ultra low volume, Biorational insecticides, Choice experiments, Knock down, Probit analysis, IPM

## Abstract

A prototype olfactory device was developed and used for first time to study the bioactivity of Ultra Low Volumes (ULV) of three essential oilsagainst the moth pest *Anarsia lineatella* (Lepidoptera: Gelechiidae). Particle sizes calibration and standard ULV time-doses range tests were performed prior the olfactory bioassays. Three essential oils were tested *Cymbopogon citratus* (Lemon Grass), *Gaultheria procumbens* (Winter Grass) and *Rosmarinus officinalis* (Rosmarin) according to the proposed method. The most active oil was that of *R. officinalis* and moths expressed approximately 3–5 fold faster moving behavior (50% repellence response times to ULV, RT50: 20–30 min) compared to *G. procumbens* (RT50:74–79 min) and *C. citratus* (RT50:82–96 min). Apart from direct observed repellence, moths sprayed with ULV show clearly signs of knock down symptoms and high fatality in a period 15–60 min after their treatment especial in the case of *R. officinalis*. Longevity of female moths was significantly affected by the initial ULV application. Furthermore, choice test showed that essential oils significantly deterred oviposition in most cases. Considering the urgent need for alternative to conventional pesticides the current work may provide a framework of testing the bioactivity of bio rational compounds in the form of ULV and under Lab conditions.

## Graphical abstract

**Figure:**
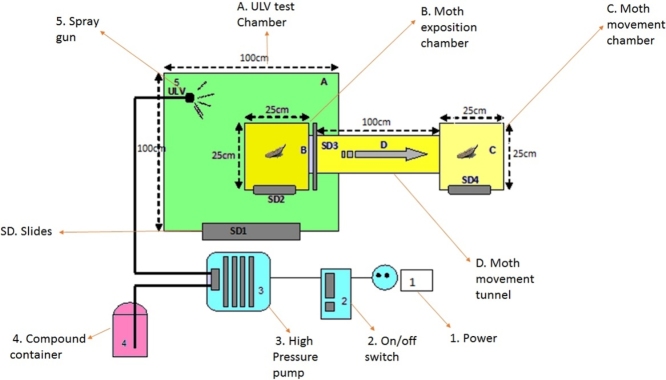

## Introduction

Lepidopteran larvae in fruit orchards are the most important pests, followed by aphids and mites. For stone fruits particularly the peach twig borer *Anarsia lineatella* Zeller (Lepidoptera: Gelechiidae) is considered as major economic pests in central and southern Europe [1]. Its distribution, habits, and damage to various hosts is discussed by Bailey, Summers [2,3], while its biology and specific life cycle traits have been extensively studied by Damos and Savopoulou-Soultani and Damos [4,5]. However, since the damage potential of *A. lineatella* in peach orchards is very high, much time and effort has been spent during the last years on developing and applying Integrated Pest Management (IPM) programs and improve rational control [6].

IPM in fruit orchards is a decision-based process, involving coordinated use of multiple tactics for optimizing the control of all classes of pests (insects, pathogens, vertebrates and weeds) in an ecologically and economically sound manner [[7], [8], [9]]. Most IPM programs use current comprehensive information on the life cycles of *A. lineatella* and their interaction with the environment (i.e. phenological models, thresholds) and are an essential component of Integrated Fruit Production (IFP) [10]. The IFP framework provides an economical and high quality fruit production, giving priority to ecologically safer methods, minimizing the undesirable side effects and use of pesticides, to enhance the safeguards to the environment and human health [7].

Since IPM uses all available means to maintain pest populations below levels that would cause economic loss while minimally impacting the environment [10], the rational use and the replacement of current synthetic insecticides with bio-rational is now a fact in most of the EU members without dispute [11]. Actually, the increasing use of bio-rational products in managing Lepidoptera in fruit orchards is the result of numerous side effects that have been raised due to the extensive use of conventional synthetic insecticides [6]. In this context, natural products are an alternative to synthetic pesticides as a means to reduce negative impacts to human health and the environment [12].

Essential oils of botanical origin are probably among the best-known substances to have attracted attention in recent years as potential bio-rational pest control agents due to their insecticidal, repellent and/or antifeedant properties [[14], [15], [16]]. Therefore, many studies have been undertaken to establish new control practices with lower mammalian toxicity and low persistence in the environment and especially using essential oils [17,18]. However, most are volatile and can act as fumigants, thus offering the prospect of use as repellent mostly against stored pest species [13,19]. Moreover, due to the low negative side effects on humans, essential oils have been evaluated to repel vector borne diseases [20]. As a result, most bioassays were done for Diptera species, in particular those ones belonging to the genus *Aedes*, *Anopheles* and *Culex* [[21], [22], [23], [24]] and which are related to diseases of public health concern such as malaria, yellow fever, dengue and viral encephalitis. Nevertheless, because stylization, application and persistence of essential oils is fraught with difficulties, bioactivity studies on other species, including agricultural pests [[25], [26], [27], [28], [29]] and particularly Lepidoptera pest threats, have received less attention.

Recent evaluations of Ultra Low Volume (ULV) and thermal fog application have been reported as effective means to apply repellents in different environments and particularly against mosquitoes [30]. Among the main advantage of the ULV application are: shorter application time due to higher flow rate (liter/hour), lower concentration of active ingredient (lower hazard). According to the U. S. Environmental Protection Agency ULV as used in common agricultural practice for instance refers to a total volume of 0.5 gal or less per acre (∼1.89 L per acre, or ∼4.67 l/ha) broadcast.

These features, along with additional benefits of minimized operator and environmental contamination, may have important implications in the context of an IPM program. Nevertheless, most ULV applications applied in pest management are using special oil-based of conventional pesticide formulations and although bio-rational compounds are potentially important elements in plant protection and in any IPM program [[31], [32], [33], [34]] there is not much evidence (either in lab or field) of the potential bioactivity of natural compounds and particularly the use of ULV of essential oils against moth species.

The objective of this study was to evaluate *in vitro* repellency and oviposition deterrence effects of three essential oils, including *Gaultheria procumbens* (winter green), *Cymbobogon citratus* (lemon grass) and *Rosmarinus officinalis* (Rosemary) against *A. lineatella* moths. These plant extracts have traditionally attracted most research duo to their high bioactivity on other species and where therefore selected to be tested as potential repellence and oviposition deterrence compounds. Moreover, the recent researcher of ULV aerial sprays, for rational pest control, provided the stimulus for the design and conduction of prototype ULV olfactory experiments instead of simply use of essential oil vapours that are used in traditional studies.

## Methods

### Moth population

A colony of *A. lineatella* was established in the Laboratory of Applied Zoology and Parasitology in the Aristotle University of Thessaloniki (Thessaloniki, Greece), from field-collected larvae present on infested twigs and peaches in northern Greece and as described by Damos and Savopoulou-Soultani [4]. The larvae were reared on artificial medium [[35], [36], [37]] and all stages maintained at rearing chambers at constant laboratory conditions at 25 ± 1 °C, 65 ± 5% RH, and a photoperiod of 16:8 (L:D)h. Adults were sexed and maintained insight plastic truncated conical caps (5 by 7.5 by 9.2 cm), covered by a transparent plastic sheet before treatment. A hole in the bottom of each cup was punched and plugged with dental roll wick providing adults with 10% sucrose solution.

### Essential oils

Three essential oils were used for the bioactivity studies: *Gaultheria procumbens* (*Winter Grass*), *Cymbopogon citratus* (Lemon Grass) and Rosmarinus officinalis (Rosemary) of 97% purity (Sigma-Aldrich, Chemical Co., USA). Essential oils were diluted in distilled water at 10% (v/v) concentration, plus Tween-20 (Amersco) (0.5%) before tests. After the dilution all essential oil mixtures were stored in a refrigerator at 4 °C until being used in the treatments. Analytical standard of Soybean oil (Sigma-Aldrich, Chemical Co., USA) was used as base for the negative controls [32].

### Olfactometer and ULV in vitro bioassays

**Fig. 1:**
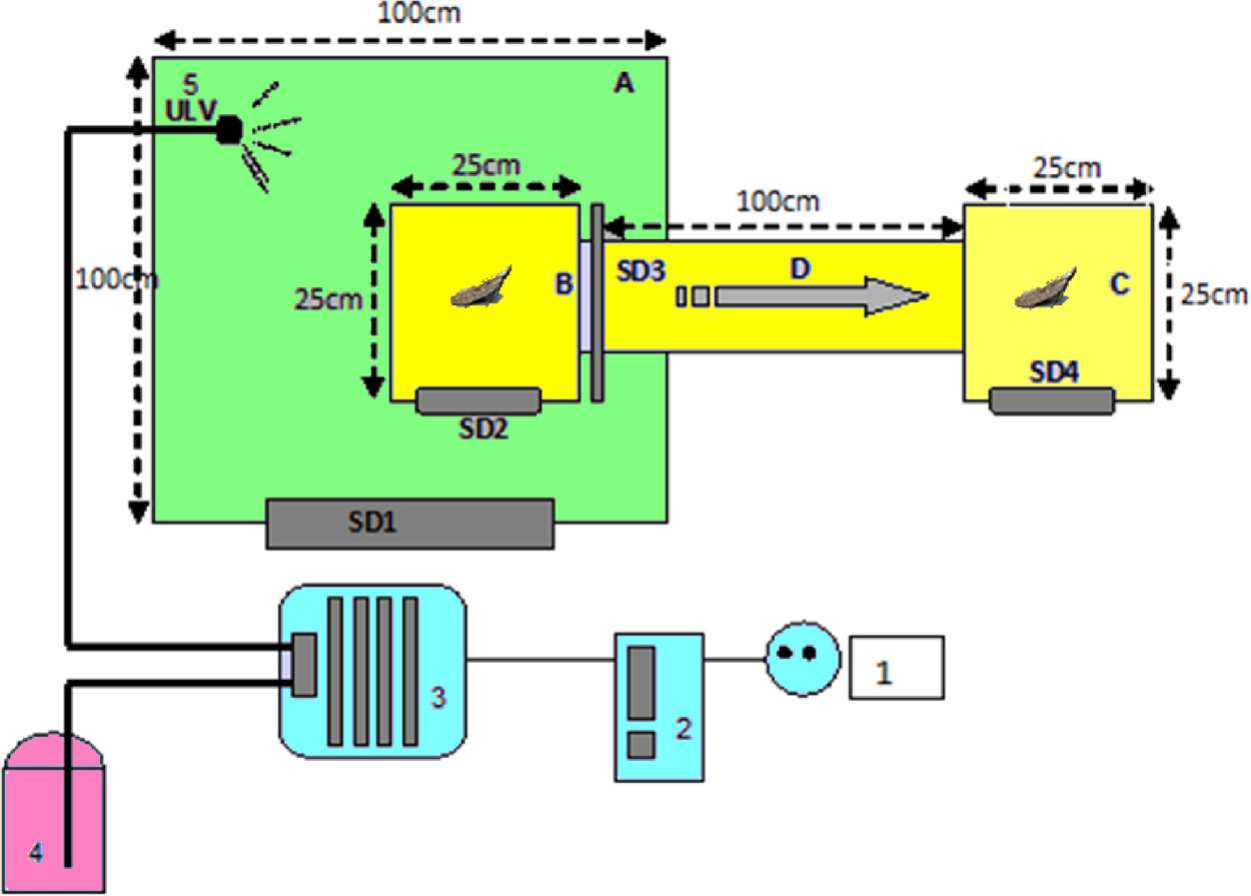
Design of ULV-olfactory apparatus for the evaluation of essential oils bioactivity and repellency against the fruit moth *A. lineatella* (vertical view) (A) ULV test chamber; (B) small moth chamber with vertices of copper wire mesh and right sliding door; (C) chamber connected with tunnel for repellency studies; and (D) tunnel; (SD) Slight doors; 21 × 21 cm (1) power; (2) on/off switch and time spray control buttons; (3) high pressure pump (4) containers of essential oil extracts (5) ULV spray gun.

For the repellent bioassays a prototype ULV devise was developed. Repellency of oil extracts against *A. lineatella* was evaluated using the olfactometer ULV device of Fig. 1. The apparatus consist of a modification of the device of Tripathi et al. [33] used for the study of repellent activity of piperitenone oxide vapors against *Anopheles stephensi* (Diptera: Anophelinae) as well as for other mosquito species. The major differentiation is the incorporation of the adaption of an ULV blower-pressurized sprayer system.

The system consists of customized electric single cylinder piston compressor 1/6 hp, with air 3.0 L air tank, a net weight of 8.4 kg. The dynamic working pressure of the instrument ranged from 0 to 4 bar (58 psi). Moreover, the apparatus has adjustable nozzles producing spray droplets ranging from 0.2 mm to 1.0 mm. The devise is connected to an automatic time switch to set ULV application rates (ranging from 1 s to 10 min). The total volume, *V*, of an essential oil solution sprayed into the actual space for a particular time, *T*, is:

(1) V=Q∙T

Where *Q* is the flow rate; which is can be further estimated by knowing the nozzle diameter, *D* and the dynamic work pressure, *P*, of the apparatus:

(2) $$Q=28.9∙D^{2}\cdot \sqrt{P}$$

In principle the device consist of two cages, B and C, which are connected through a tunnel D. The cage B is enclosed insight a larger plastic chamber A in which the ULV particles are sprayed through a 0.8 mm droplet nozzle. For the repellency and bioactivity studies 10–20, 3 days old, male and female moths were placed insight the cage B and treated with ULV of essential oils for certain times. Preliminary trials were carried out; first to measure the mean droplet size produced using the ULV customized devise, as well as the effect of pressure on droplet size distribution and then, to estimate the mean ULV spray application times that may affect moth movement. Thus, based on these trials, moths were subjected to ULV treatments for two time intervals, for 5 and 10 s, using an 0.8 mm in diameter nozzle and 2 bar pressure (see below), which corresponds to ULV volumes of 63.09 cm^3^ and 126.18 cm^3^ for each time treatment respectively.

### Particle sizes calibration and standard ULV time-doses range tests

The median diameter of all the particles collected on single slides by gravitational settling was determined by calculating the volume of each droplet. Microscope slides were placed in the center of the ULV-olfactory device and treated with essential oils for 5 s. The ULV droplets that have been deposited 5 min after treatment on the slide were placed under a microscope to measure diameter of individual droplets with an eyepiece micrometer. All particles that were deposited from the one side of the slide to the other and that passed through the micrometer scale, as the slide was moved, were measured. Measurement was avoided in cases in which particles of the smaller size groups were congregated along the margin of the slide and were difficult to be counted as separate. The number of the particles that were deposited on the slide were measured and clustered in 12 ranks-classes according to their diameter and compared, for confirmative reasons, with water sensitive cards of standard droplet deposition (Courting spraying systems Co, Syngenta Crop Protection, 2002). The diameter of the particles was measured in microns (1 μ  = 1 μm = 1/1000 mm = 10^−6^ m). The effect of pressure on the particle diameter was examined using four flow rate- pressure settings: 1/4, 1/2, 3/4, and for a standard 0.8 mm nozzle.

The initial application times of the standard essential oils concentrations were chosen based on range–finding tests, to cause repellence between 10 and 90%. For each of the essential oils the time – doses of 1, 5, 10, 15 and 30 s were tested; each with three replicates and thirty individuals per each replicate.

### Moth repellence, knock down and mortality bioassays

For the repellency studies the criterion was migration of moths from chamber B to C via the tunnel D after 1, 5, 15, 30, 60 and 120 min. In addition, the bioactivity of the selected essential oils against fruit moth was further evaluated by considering the number of knocked downed and dead moths registered in chamber C at the previous time intervals. Bioassays were based on dose-response trials and probit-analysis (see below). In this approach the biological responses of interest is plotted against different doses of the same causal stimuli (or logarithms of them) and by taking into account the natural response rate (i.e. zero-dose using the same solvent). For each essential oil treatments were performed and at least five replications. For the record, although knock down and mortality evaluation are usually tested under conditions that are not repellent this option was not chosen because the current work aimed to evaluate both; the repellent activity of the essential oils as well as any latent adverse effects that are observable short time after the moth exposure to the ULV. Additionally, such an approach is considered to be closer to field conditions. Particularly, it is of interest not only if an insect is repelled after the application of a bioactive compound, but also, if it is lethal affected short time later after its movement.

### Oviposition deterrence choice tests

The effect of the three essential oils on oviposition and egg hatching of *A. lineatella* was studied by introducing individuals from the laboratory colony in plastic oviposition cages (23 × 23 × 23 cm) under choice and non choice conditions.

Plant material consisting of peach twigs having one fruit and 2–5 leaves (∼5–10 cm) served as oviposition substrate. The plant material was plugged by the same manner as previous described for moth rearing, in two small circular plastic cups (3 by 3 cm) with dental roll wick providing adults with 10% sucrose solution. The plant material was treated with standard ULV 15 s time-dosages of standard concentrates using the olfactometer device. Plant material sprayed with distillated water, soybeen oil and emulsifier (Tween 20) served as untreated check. All ULV treated oviposition substrates were air dried for 15 min and then placed insight the oviposition cages.

In the choice test, treated and untreated plant material was placed in the same plastic cages, 32 cm apart, to provide a choice for oviposition. Cages were effectively split into two, with a ‘treated’ half and a ‘control’ half. The individual oil treated oviposition substrates were assigned at random to 30 cages (equivalent to main plots) so that there were at least five to ten replicates per treatment. In the no-choice test, the treated and untreated fruits were placed in different cages, each containing 2–5 females. The experiment was arranged according to a randomized complete block design. The numbers of eggs laid per female per 24 h, and survival of females, were recorded. Experiments were terminated when all females died.

## Data processing and statistical analysis

Prior any data analysis the percentages of time responses (e.g. repellency, knockdown effects and mortality) were corrected for the natural response rate by using Abbott's calculation formula as follows [38,39]: Corrected % response = (% response in test − % response in control)/(100 − % response in control) × 100. To meet normality, which is recommended particularly for parametric mean comparisons and analysis of variance (ANOVA) [40,41], data were transformed using arcsine √x transformation [42].

### Volume median diameter (VMD) of droplets and frequency distribution

The calculation of median droplet diameter was based on the definition of moments of a distribution. Since the relationship between droplet diameter (or volume/time) and pressure (PSI) in not linear, an exponential probability function was used to describe VMD frequency distributions, as follows [[43], [44], [45]]:

(3) $$f\left(x;\lambda \right)=\left\{\begin{array}{c} \lambda \;e^{-\lambda x},x\geq 0\\ 0\;,\;x<0 \end{array}\right),$$

Having $\mu =\lambda^{-1}$mean: λ^−1,^ median λ^−1^ ln2 and variance λ^−2^, with rate parameter λ > 0.

The following power law, empirical regression function, was fitted on data to extrapolate the effects of pressure on particles sizes:

(4) $$f\left(x;a.k\right)=ax^{k},$$

where *α* and *k* are calibration parameters. The goodness-of-fit, of the pressure related multiple distributions for the given data set, were compared according to the adjusted Anderson-Darling statistic [46]. The statistic is:

(5) $$AD^{*}=n\sum_{i=1}^{n+1}(a_{i}+b_{i}+c_{i}),$$

where: $\;a_{i}=-z_{i}-\ln\left(1-z_{i}\right)+z_{i-1}+\ln\left(1-z_{i-1}\right)$, $\;b_{i}=2\ln\left(1-z_{i}\right)f_{i}(z_{i-1})-2ln(1-z_{i-1})f_{n}(z_{i-1})$, $\;c_{i}=\ln\left(z_{i}\right)f_{n}{\left(z_{i-1}\right)}^{2}-\ln\left(1-z_{i-1}\right)f_{n}{\left(z_{i-1}\right)}^{2}-\ln{\left(z_{i-1}\right)}^{2}+\ln\left(1-z_{i-1}\right)f_{n}{\left(z_{i-1}\right)}^{2}$ and *z_i_* is the fitted estimate of the cumulative distribution function, *cdf*, for the ith plot and f_n_(*z_i_*) is the non-parametric estimate. Distribution with the smallest adjusted Anderson-Darling statistic offers the best fit [[47], [48], [49]]. Goodness of fit for the power law regression was evaluated by estimating the coefficient of determination *R*^2^.

### Bioactivity and time response bioassays probit analyses

Firstly, preliminary bioassay tests were carried out to determine the effective time application of standard ULV concentrations for each treatment. Five application times (1, 5, 10, 15 and 30*sec*) were tested for each essential oil and were subjected to probit analysis. After the estimation of the effective time range, two representative ULV application times were selected (5 and 15 s) and separate bioassay tests were carried out to determine bioactivity (repellence, knock down and mortality) and estimate mean time responses of the moths to essential oils treatments. In particular, the data that were obtained from each time-response bioassay were subjected to probit analysis using the following probit analysis algorithm [42].

Data were aggregated for every covariate pattern and response data considered as binary (success or failure), so that each response represents a random realization which is described according to a binomial distribution:

(6) $$\begin{array}{cc} P\left(x_{i}=r_{i}\right)=\left(\begin{array}{c} n_{i}\\ r_{i} \end{array}\right)P_{i}^{r_{i}}{\left(1-P_{i}\right)}^{\;n_{i}-r_{i}}, & i=1,2,\ldots ,n, \end{array}$$

where: *n_i_* and *r_i_* represent the number of subjects and responses for *i*-th of the *m* covariate pattern, respectively.

The log likelihood *L* for *m*observations, after ignoring the constant factor, is:

(7) $$L=\sum_{i=1}^{m}r_{i}\text{ln}P_{i}+\left(n_{i}-r\right)\ln\left(1-P\right).$$

For the time (or dose) - response models it was further assumed that:

(8) $$P_{i}=\gamma +\left(1-\gamma \right)F\left(X_{i};\beta \right),$$

where, *γ* represents the natural response rate and *X_i_* is a *n *× (*p *+ *q*) matrix with element *x_ij_*, which represents the *j-*th covariate for the *i-*th covariate pattern for p number of independent variables and *q* number of levels of the grouping variable.

The related to the probit model cumulative distribution function *F*(X*_i_* ;β) equals:

(9) $$F\left(X_{i};\beta \right)=\int_{-\infty }^{X_{i}\beta }\left(\frac{1}{\sqrt{2\pi }}e^{-z^{2}\frac{1}{2}}\right)dz,$$

and *β* is a (*p *+ *q*) × 1 vector which is a composite of independent variable of *γ* and *α*. Finally, all probit transformed data were regressed against log10-transformed response times.

Goodness of fit for each probit model was estimated according to the Pearson chi-square statistic as follow [45]:

(10) $$x^{2}=\sum_{i=1}^{m}[\;{\left(r_{i}-\hat{E_{\iota }}\right)}^{2}/\hat{E_{i}}\left(1-\hat{P_{i}}\right)$$

Where $\hat{E_{i}}=n_{i}\hat{P_{i}}$ is the expected frequency and $\hat{P_{i}}=\hat{\gamma }+\left(1-\hat{\gamma }\right){\hat{F}}_{i}$. Response times were expressed as time values RT0.5 s, RT10s and RT50s and 95% fiducially limits for each case were generated [42,50].

### No choice test data analysis

Non choice tests were designed to evaluate the oviposition preference of the moths with a single test oviposition substrate, for a fixed period of time, in plastic cages under laboratory conditions and as described in the oviposition deterrence choice tests section [[51], [52], [53]]. ANOVA and Duncan’s multiple range tests were further employed on oviposition data; using SPSS 9 software for Windows to compare means [42,54,55]. The ANOVA *F*-statistic is the ratio of two independent chi-square variables divided by their respective degrees of freedom was estimated as:

(11) $$F=\frac{\sum n_{j}{\left(x_{j}-\bar{x}\right)}^{2}/\left(k-1\right)}{\sum \sum {\left(x_{ij}-\bar{x}\right)}^{2}/\left(n-1\right)},$$

Where *k-1* stands for the degrees of freedom between groups and *n-1* are the total degrees of freedom. The simple-article hypothesis is that all sample means are the same and was rejected for a $F_{0.05;k-1;n-k}$. Duncans significant difference is given by [54]:

(12) $$R_{p}=r_{0.05,p,\nu }*SE_{d}/\sqrt{n},$$

where $r_{a,p,v}$ is the *Duncan’s Significant Range Value* with parameters for *α* = 0.05 significance level, *p = 2,…,n* is the distance in rank betweens pairs of multiple comparisons and *v *= Mean square errors degree of freedom.

For comparative reasons, the non-parametric equivalent to ANOVA test of Kruskal-Wallis was also performed [56,57]. The probability distribution and the related statistic is given by:

(13) $$H=\left(n-1\right)\left[\sum_{i=1}^{k}n_{i}{\left(\bar{r_{i}}-\bar{r}\right)}^{2}/\sum_{i=1}^{k}\sum_{j=1}^{n_{i}}n_{i}{\left(\bar{r_{ij}}\;-\bar{r}\right)}^{2}\right],$$

where:

*n* number of observations across all groups, *n_i_* number of observations in group *i*, *r_ij_* rank of observation *j* from group *i*, $\bar{r_{i}}=\sum_{j=1}^{n_{i}}r_{ij}/n_{i}$ and $\bar{r}=\left(N+1\right)/2$. The *p* value is approximated by a chi-square distributed $\text{Pr}\left(x_{k-1}^{2}\geq K\right)$ and simple-article hypothesis that of median equality was rejected if $H\geq x_{0.05;k-1}^{2}$.

### Two choice tests data analysis

Hypothesis testing about the moth oviposition preference in the two-choice experiments was analyzed using classical nonparametric methods in which for each individual the observations consists of the number of times choice 1 (or choice 2) is preferred to lay eggs [58]. Data were examined to verify whether they met the assumptions of normality, and all statistical comparisons were made using two-sample *t*-tests. The hypothesis of *H_0_:m_1_-m_2_* *= d_0_* versus *H_1_:m_1_-m_2_* *≠ d_0_*, where *m_1_* and *m_2_* are the population means and *d*_*0*_ is the hypothesized difference between the two oviposition means, was tested for α = *0.05* level of significance [42]. The *t*-statistic is calculated as:

(14) $$t=(\bar{x_{1}}-\bar{x_{2}}/\sqrt{\{\left[{\left(n_{1}-1\right)}^{2}\cdot s_{1}^{2}+{\left(n_{2}-1\right)}^{2}\cdot s_{2}^{2}\right]/\left(n_{1}+n_{2}-2\right)\}\cdot [(n_{1}+n_{2})/(n_{1}\cdot n_{2}})],$$

Where, ${\bar{x}}_{1}$ and ${\bar{x}}_{2}$ are the means, $s_{1}^{2}$ and $s_{2}^{2}$ the variances for samples $n_{1}$and$n_{2}$, respectively. Critical values for the standard significance level α = 0.05 are known and *H*_*0*_ is rejected if:

(15) $$\left|t\right|>t_{N-2;a/2}$$

For comparative reasons, a two-sample non-parametric Mann-Whitney U rank test was also performed to test the hypothesis of *H_0_:n_1_* *= n_2_* versus *H_1_:n_1_* *≠ n_2_*, where *n* is the population median and to compare mean ranks [41].

The test statistic is defined below:

(16) $$U_{1}=n_{1}n_{2}+\frac{n_{1}\left(n_{1}+1\right)}{2}-R_{1}$$

(17) $$\text{and}\;\;U_{2}=n_{1}n_{2}+\frac{n_{2}\left(n_{2}+1\right)}{2}-R_{2},$$

where *R_1_* and *R_2_* denote the sum of the ranks in samples $n_{1}$ and $n_{2}$, respectively. For equal populations we expect *R_1_*and *R_2_*to be similar. The smaller value *U*_i_ of *U*_1_ and *U*_2_ is then used to consult significance tables. The theoretical range of *U*_i_ is from 0 (complete separation between groups; *H_0_* most likely false and *H_1_* most likely true) to n_1_*n_2_ (little evidence in support of *H_1_*). Thus, *H_0_* is rejected for low values of *U*_i_.

## Results

### Volume median diameter (VMD**)** probability distribution frequencies

The diameter of the drops produced, using the current equipment, ranged from 100 to 400 μm which is closer to droplet sizes of agricultural sprays [16], but higher compared to standard ULV applicators. Commercial devices may generate droplets which can vary from 38 μm to 65 μm [59]. Nevertheless, standard spreader supplied with mist sprayers have diameter average which is about 100 μm, or even more, according to the nozzle and pressure used.

**Fig. 2:**
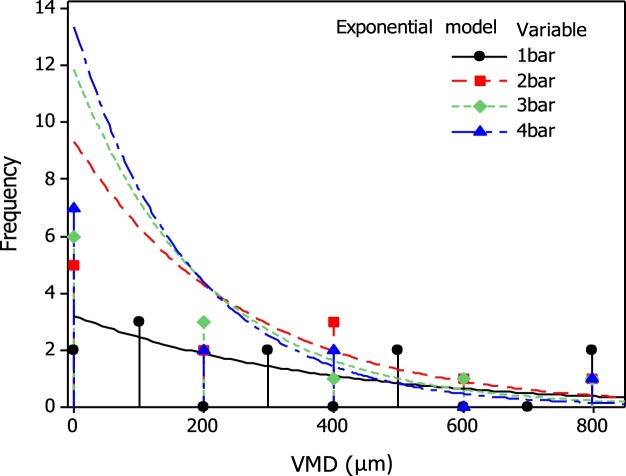
Volume median diameter (VMD**)** exponential frequency distributions of the fine droplets that were settled on the microscope slides using the ULV – olfactory apparatus under different bar pressures (variables). Dots represent counts and line the fitted exponential model. All Measurements were performed using standard nozzle 0.8 mm in diameter.

**Fig. 3:**
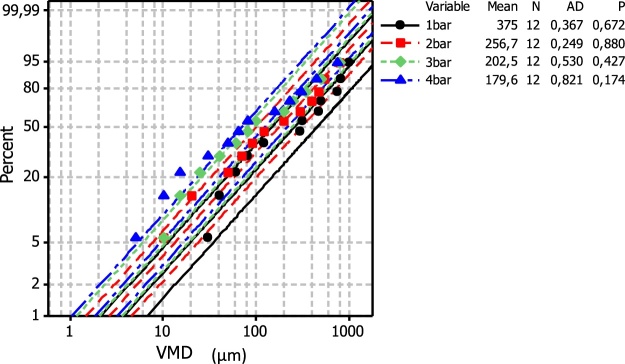
Volume median diameter (VMD**)** probability distribution frequencies of the fine droplets that were settled on the microscope slides using the ULV – olfactory apparatus under different bar pressures (variables). Dots represent counts and line the fitted log transformed exponential model. All Measurements were performed using standard nozzle 0.8 mm in diameter. Mean: *λ^−1^*, *N*: number of droplets classes, *AD*: Anderson-Darling statistics and the corresponding *p*-value for each distribution (p-values greater than *α:0.05* suggests that the data follow the exponential distribution).

The size of volume of the drops arising from each pressure is been shown to follow an exponential distribution: thus the mean and median diameter is quoted by the parameter *λ* and specifies the distribution for each case (Fig. 2, Fig. 3). In all cases the particle diameter frequencies are well described according to the exponential model which provides good fits (Fig. 2) as well as the related log transformed percent probability plots (Fig. 3).

**Fig. 4:**
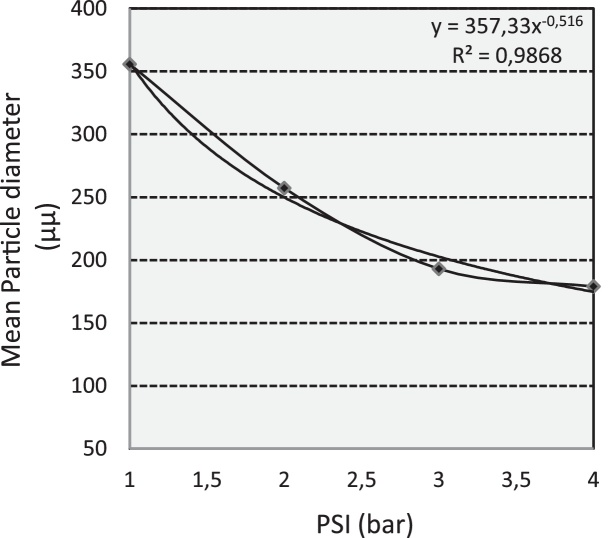
The effect of pressure on droplet size settled on the microscope slides using the ULV – olfactory apparatus and described according to the power law regression.

Because the spray solution emerges from the nozzle in a sheet, and droplets form at the edge of the sheet, as spray pressure increases the sheet becomes thinner, it probably breaks into smaller droplets than from a sheet produced at lower pressure. Thus, the diameter of the spray droplet is inverse related to spray pressure (Fig. 4) and this trend is well captured by the power law regression model that was fitted on data (R^2^ > 0.9). According to the power law model and for a standard nozzle diameter (0.8 mm) to compensate decrease in volume size as soluble flow increases, moderate pressures of 2 bar produce 250 μm droplets. However, there are no considerable differences when pressure increases from 3 to 4 bars and produced droplets have approximately the same VMDs.

### Median ultra low volumes ULV_50_

**Fig. 5:**
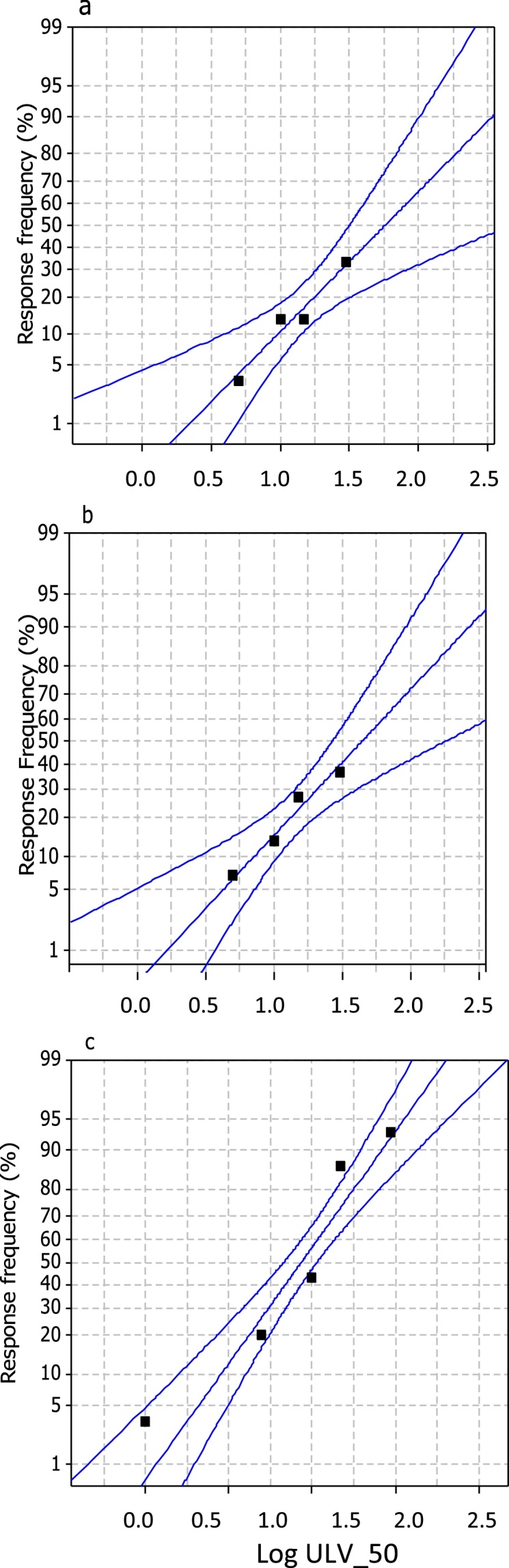
Probit transformed response frequency expressed as percentage deterrence in relation to standard log-transformed ULV application times for *C. citratus* (a), *G. procumbens* (b) and *R. officinalis* (c) (dots out of the scale are not plotted). In all cases, standard 10% dilutions (v/v) of essential oil concentrates were used for the ULV treatments and ULV_50_ represents the median of the moth tolerance distribution against essential oils.

The *ULV*_50_ moth deterrence was expressed as percentage deterrence in relation to standard log transformed ULV application times for each case (Fig. 5a–c, respectively). Fitting of the probit transformed model is considered only on normal equivalent deviate and without adding 5. Therefore, according to the probit model the log ULV_50_ corresponds to the value of probit = 0 and the observed probits which are plotted against lie almost on a straight line. Alternatively, the ULV_50_ values can be regarded as the median of the tolerance distribution which was used to evaluate the actual level of tolerance such that the half moths lie on either side of it. These bioassay tests indicated that all treatments, except control, caused repellence to *A. lineatella* moths’ very short time after their application (5 s). However, *R. officinalis* (Log ULV_50_:0.938; 95%CI: 0.843–1.034; P = 0.029; DF = 3; *x^2^*: 9.02136) caused considerably higher deterrence than those caused by *G. procumbens* (Log ULV_50_:0.751; 95%CI: 0.45–1.05, P = 0.981; DF = 2; *x^2^*:0.3888) and *C. citratus* (LogULV_50_: 0.788, 5%CI: 0.525–2.513, P = 0.988; DF = 3, *x^2^*:0.1329).

### Repellence median time responses – RT_50_

Probit analysis data concerning repellent activity of essential oils on *A. lineatella* moths and for two different ULV application times are given in Table1. These values represent the median response times to the half of the moth population when treated with standard ULV of essential oils and is been used to estimate more precisely how the stimulus has been perceived by the moths. The median response times RT_50_ for moths treated with ULV essential oil for certain time differ for each case. As in the case of the dose response bioassays, migration time of moths from the treatment chamber B, to the clear chamber C via the tunnel were shorter for cohorts that were exposed for longer time with essential oils ULVs. For example, half of the individuals that were treated for 15 s with *R. officinalis*, migrated after half an hour (21.099 and 32.688 min, for male and females respectively), while moths treated for 5 s migrated after one hour (65.832 min and 67.306 min, for males and females respectively). Such patterns were also observed when the moths treated with the other two essential oils, although individuals remained for longer times in the treatment chamber before migrating. It also is noteworthy to indicate that in some cases (i.e. *G. procumbens* and *C. citratus* on the 15 s treatments), probit analysis produced low-value slopes due to some infrequent observations (outliers) and no confidence intervals, although *x^2^* was quite high suggesting good model fit. Finally, there were no significant differences concerning moving behavior between male and female moths.

**Table 1:** Probit transformed time responses of *A. lineatella* to repellent activity of essential oil ULV treatments resulting from in vitro olfactory trials.

				Probit Analysis				
						95% CI		
*Treatment*	*Essential oil*	*sex*	*N*	*slope*	*RT_50_*^a^	*Lower*	*Higher*	*x^2^*
*5 s ULV*	*Gaultheria procumbens*	♂♂	30	0.023	109	75.147	389.352	11.815
		*♀♀*	35	0.018	136.735	86.670	1312.853	13.715
	*Cymbopogon citratus*	*♂♂*	15	0.014	110.029	–	–	11.629
		*♀♀*	19	0.010	158.441	–	–	11.765
	*Rosmarinus officinalis*	*♂♂*	18	0.028	65.832	50.156	109.334	8.494
		*♀♀*	19	0.025	67.306	50.085	120.093	11.260

*15 s ULV*	*Gaultheria procumbens*	*♂♂*	15	0.027	82.385	–	–	23.452
		*♀♀*	23	0.022	96.591	67.207	276.922	10.920
	*Cymbopogon citratus*	*♂♂*	15	0.031	74.360	55.708	152.471	4.182
		*♀♀*	15	0.027	79.208	59.825	145.541	17.035
	*Rosmarinus officinalis*	*♂♂*	19	0.041	21.099	4.359	43.539	39.563
		*♀♀*	16	0.040	32.688	20.461	54.669	24.476

a RT_50_: 50% repellence response times to ULV of essential oils are expressed in min after application and are considered significantly different when 95% fiducial limits fail to overlap.

### Knock down median time responses – KD_50_

Table 2 presents the probit analysis data concerning the median times at which moth knock down responses were observed after the ULV treatments. According to the *x^2^* values of the Pearson statistic the knock down moth responses to ULV exposure was captured well by the probit transformed models and especially for *R. officinalis* and *G. procumbens* (Table 2). Moreover, knock down of moths was observed after the passage of half to one hour after ULV application. These backward insecticidal effects were observed in all cases, but the 5 s time –dosage application of *G. procumbens*. In most cases male and female moth responses showed very little variation between them and 95% fiducial limits fail to overlap. The knock down effect caused by *R. officinalis* was higher (KD_50_: 62.279 and 78.914 min, for male and females) compared to *C. citratus* (KD_50_: 77.514 and 82.468 min, for male and females) and *G. procumbens* (KD_50_: 100.089 and 109.452 min, for male and females).

**Table 2:** Probit transformed time responses of *A. lineatella* to knock down of essential oil ULV treatments resulting from in vitro olfactory trials.

			*Probit Analysis*					
						*95% CI*		
*Treatment*	*Essential oil*	*Sex*	*N*	*slope*	*KD_50_*^a^	*Lower*	*Higher*	*x^2^*
*5 s ULV*	*Gaultheria procumbens*	*♂♂*	30	–	–	–	–	–
		*♀♀*	35	–	–	–	–	–
	*Cymbopogon citratus*	*♂♂*	15	0.028	96.240	–	–	6.406
		*♀♀*	19	0.033	66.615	51.291	113.322	6.788
	*Rosmarinus officinalis*	*♂♂*	18	0.049	31.444	19.081	406.459	7.148
		*♀♀*	19	0.017	62.875	42.093	171.799	14.147

*15 s ULV*	*Gaultheria procumbens*	*♂♂*	15	0.027	100.089	–	–	7.205
		*♀♀*	23	0.026	109.452	–	–	6.193
	*Cymbopogon citratus*	*♂♂*	15	0.017	77.514	49.176	459.204	8.912
		*♀♀*	15	0.021	82.468	50.856	813.741	5.785
	*Rosmarinus officinalis*	*♂♂*	19	0.021	62.279	39.146	320.971	6.387
		*♀♀*	16	0.006	78.914	–	–	11.159

a KD_50_: 50% median knock down time response to ULV of essential oils are expressed in min after application and are considered significantly different when 95% fiducial limits fail to overlap.

### Lethal median times responses – LT_50_

In this trial I have considered the individuals that either died short time after the ULV application or were able to migrate and were knocked down few minutes later and were not able to recover. The calculated lethal response times, LT_50_, to ULV treatments, 95% confidence limits and probit transformed regressions data are presented in Table 3. Both, *R. officinalis* and *G. procumbens*, had high insecticidal properties in contrast to *C. citratus* which had not adverse effect on moths. Moreover, moths lived shorter period of time when treated with *R. officinalis* (LT_50_: 14.026 and 36.548 min, for male and females), compared to those treated with *Gaultheria procumbens. (*LT_50_: 90.610 and 59.783 min, for male and females). A slight increase of susceptibility in adults with increasing ULV time doses was observed for *R. officinalis*, while no significant differences were recorded for *C. citratus*. It is worthy to state that in most cases *R. officinalis* caused direct mortality short time after its application, while *C. citratus* mostly displayed an indirect mode of action since moths firstly were knocked downed and some of them failed to recover. No significant differences were recorded between the two sexes.

**Table 3:** Probit transformed time responses (mortality) of *A. lineatella* to insecticidal activity of essential oil ULV treatments resulting from in vitro olfactory trials.

			*Probit Analysis*					
						95% CI		
Treatment	Essential oil	Sex	N	slope	LT50^a^	Lower	Higher	*x*^2^
*5 sec ULV*	*Gaultheria procumbens*	*♂♂*	30	–	–	–	–	–
		*♀♀*	35	–	–	–	–	–
	*Cymbopogon citratus*	*♂♂*	15	0.028	68.650	50.809	131.354	9.525
		*♀♀*	19	0.018	75.544	48.258	326.340	8.155
	*Rosmarinus officinalis*	*♂♂*	18	0.015	59.901	37.976	240.995	10.247
		*♀♀*	19	0.014	74.622	46.936	427.016	17.063

*15 sec ULV*	*Gaultheria procumbens*	*♂♂*	15	–	–	–	–	–
		*♀♀*	23	–	–	–	–	–
	*Cymbopogon citratus*	*♂♂*	15	0.021	90.610	59.777	469.083	6.303
		*♀♀*	15	0.022	59.783	39.025	181.469	11.654
	*Rosmarinus officinalis*	*♂♂*	19	0.025	14.026	–	50.866	36.060
		*♀♀*	16	0.020	36.548	–	–	40.371

a LT_50_: 50% lethal time responses to ULV of essential oils are expressed in min after application and are considered significantly different when 95% fiducial limits fail to overlap.

### No choice oviposition deterrence tests

**Fig. 6:**
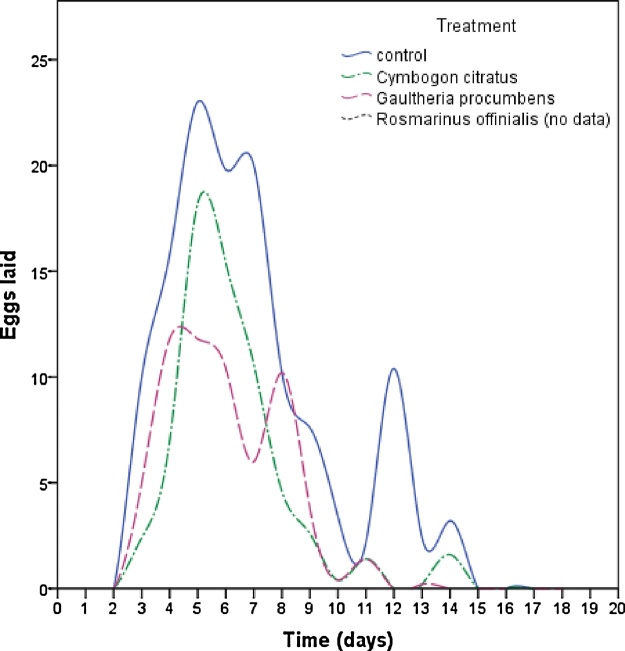
Effect of ULV of selected oils (10 mL/L) that were sprayed for 15 s on oviposition substrate, on the number of eggs/day/female laid by fruit moths (*A. lineatella*) in the non-choice test.

**Fig. 7:**
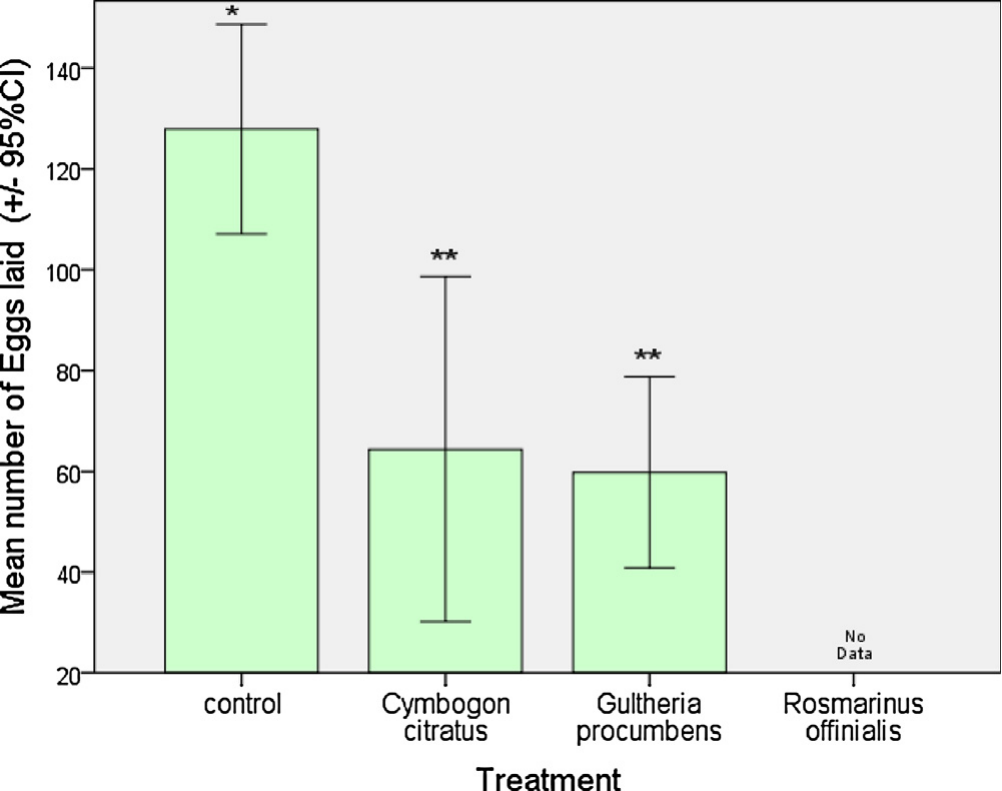
Effect of ULV of selected oils (10 mL/L) that were sprayed for 15 s on oviposition substrate, on the number of eggs that were laid by female fruit moths (*A. lineatella*) in the non-choice test. Error bars are the standard error of means. The different number of asterisks indicate multiple comparison significant differences (*P < 0.05, Duncan test).

The effect of ULV treated oviposition substrate on the female oviposition patterns are depicted in Fig. 6, while the effect on mean eggs laid is shown in Fig. 7. In most cases ULV treatments with essential oils were effective in deterring *A. lineatella* from laying eggs in fruits. However, due to the very strong insecticidal activity of *R. officinalis* and the few available replications, it was difficult to estimate the indirect effects on oviposition for this essential oil. Both essential oils, *G. procumbens* and *C. citratus*, reduced the total egg production by half compared to the control. Females laid 64.4 ± 12.3 eggs on oviposition substrate that was treated with ULV of *G. procumbens*, 59.8 ± 6.8 eggs on substrate treated with *C. citratus* and 128 ± 7.4eggs on the control. Spraying peach fruits with standard ULV (time-dose: 15 s) with essential oils, showed that the applications significantly reduced the number of eggs and according to the parametric (*F = 17.093, P = 0.000, df: 12*) and the non – parametric analysis (*x^2^ = 9.517, P = 0.009*), respectively (Fig. 7).

**Fig. 8:**
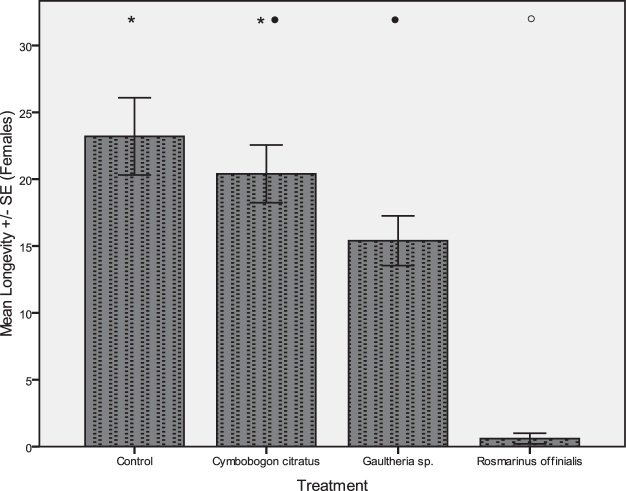
Effect of ULV of selected oils (10 mL/L) that were sprayed for 15 s on oviposition substrate, on longevity of female fruit moths (*A. lineatella*) in the non-choice experiments. Error bars are the standard error of means. The different symbols (*,●,○) indicate multiple comparison significant differences (*P *< 0.05, Duncan test).

Moreover, survivorship of female moths was also significantly affected according to the parametric (*df = 19, F = 24.379, Duncan test*) and non parametric statistical analysis (*x^2^ = 13.1991, Kruskall – Wallis test*). Thus, the female mean longevity was 20.4 ± 2.1d when they left to lay eggs on oviposition substrates that were treated with ULV of *C. citratus*, 15.4 ± 4.1 when treated with ULV of *G. procumbens*, 1 ± 0.8 when treated with *R. officinalis* and 23.2 ± 6.4d for the control (Fig. 8).

### Two choice oviposition deterrence tests

**Fig. 9:**
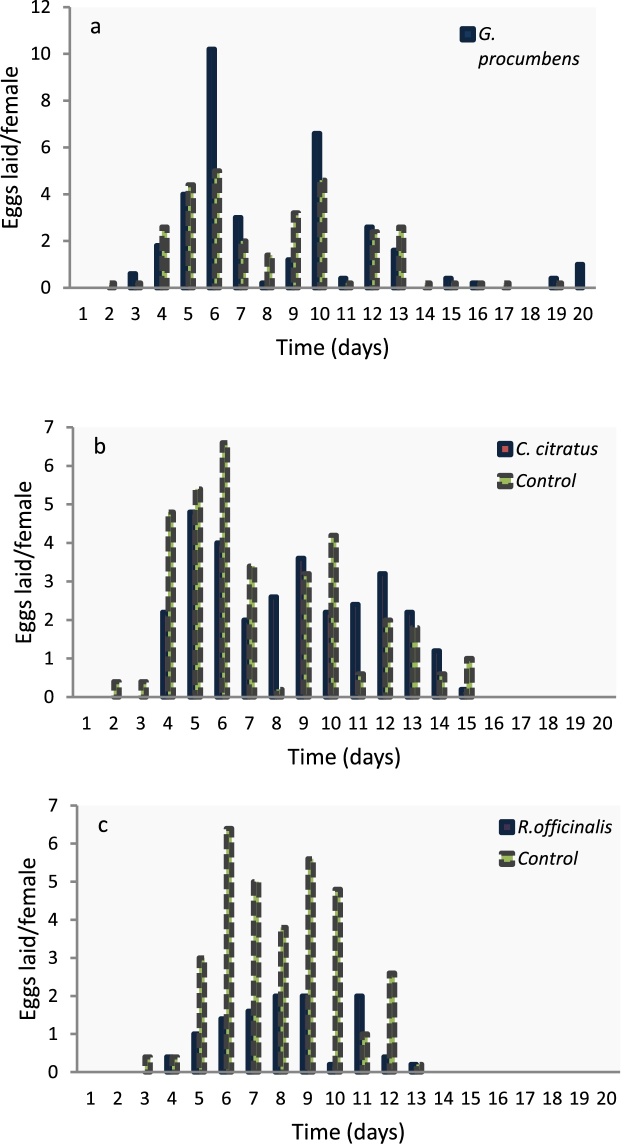
Effect of ULV of selected oils (10 mL/L) that were sprayed for 15 s on oviposition substrate, on the number of eggs laid per day by female fruit moths (*A. lineatella*) in the choice test; (a) *G. procumbens*, (b) *C. citratus* and (c) *R. officinalis.*

**Fig. 10:**
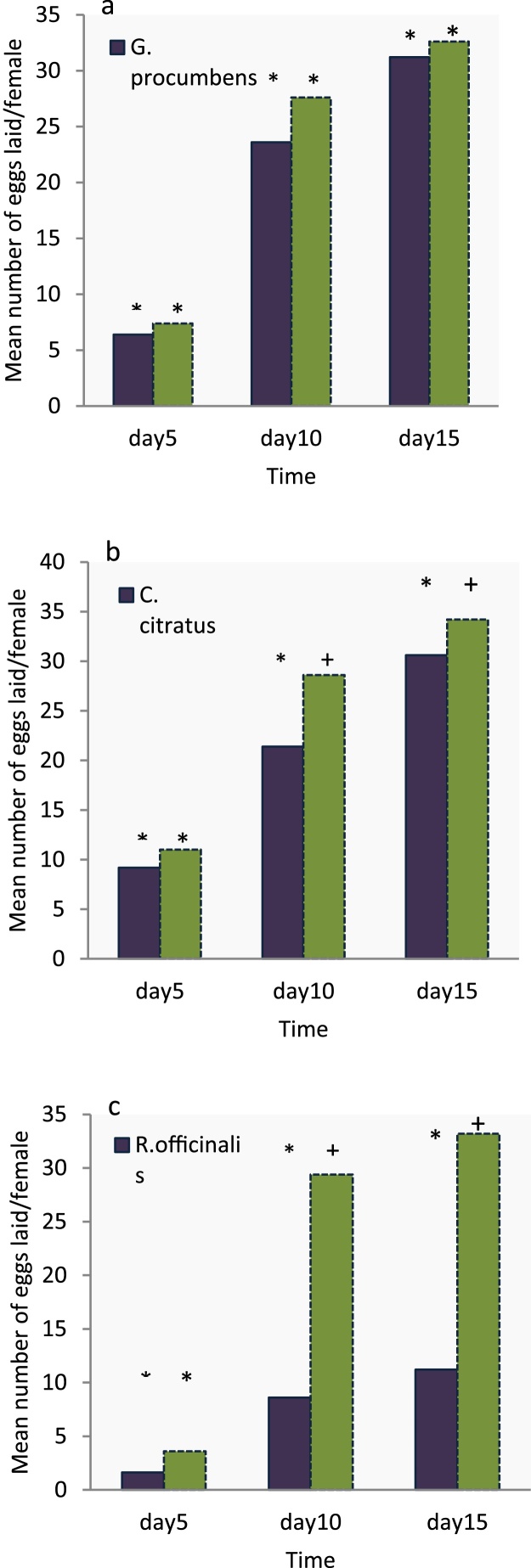
Effect of ULV of selected oils (10 mL/L) that were sprayed for 15 s on oviposition substrate, on the cumulative number of eggs that were laid at the 5, 10 and 15th days after treatment by female fruit moths (*A. lineatella*) in the choice test. The different symbols, (*,+), above treatment (dark blue) and control (green) bars, indicate pair wise significant differences (*P *< 0.05, *t*-tests).

Fig. 9 show the results of the two choice tests for the three essential oils that were tested in the form of ULV and applied for a 15 s time-dose. In most cases the essential oils had a significant effect on the number of eggs laid per day by female fruit moths (*A. lineatella*) in the choice test (Fig. 9). Fig. 10 depicts the pooled oviposition deterrence data (cumulative egg laying) that were registered after 5, 10 and 15 days of observations. Among the essential oils tested, *G. procumbens* had no adverse effects on moth fecundity at any times that were registered, while *C. citratus* and *R. officinalis* produced a significant reduction in almost all cases, with the later showing the strongest effect. Moderate adverse effects on oviposition preference were recorded for *C.citratus* and a considerable lower number of eggs were laid on the oviposition substrates that were treated, although differences were significant only for the first days of observation (Fig. 10). In particular, moths laid the same number of eggs on the treated substrate compared to the control, after 5 days (9 ± 2 and 11 ± 0.9 eggs), but not after 10days (21.4 ± 2.2 and 28.6 ± 2.6) or 15 days (30.6 ± 2 και 34.4 ± 2), respectively.

There were no significant differences on the mean number of eggs laid on oviposition substrate treated with ULV of *G. procumbens* and the control, either after 5 days (6.4 ± 2 and 7.4 ± 0.9 eggs), 10days (23.6 ± 1.5 and 27.4 ± 2.2 respectively) or 15 days (31.2 ± 2.44 and 32.6 ± 2.66), after the treatment respectively (Fig. 10). Finally, no significant differences were observed between the treated substrate with *R. officinalis* and the control after 5 days of observation (1.6 ± 1.1. and 3.6 ± 1.3), but only 10 (8.6 ± 2.8 and 29.4 ± 1.2eggs) and 15 days (11.2 ± 3.6 and 33.2 ± 1.7) after the treatments.

## Discussion

The analysis of the bioassay data showed that ULV of all three essential oils that were tested exhibit strong repellent activity against *A. lineatella* moths. In addition, the repellent activity of *R. officinalis*, was higher compared to the other two essential oils that were tested. Moths for example expressed approximately 3–5 fold faster moving behavior when treated with *R. officinalis*, compared to *G. procumbens* and *C. citratus*, respectively and for both ULV time application.

Nevertheless, a part of the repellent activity, another important consideration which brought out by the study is the substance’s possible latent insecticidal properties which are expressed in the form of knock down symptoms and latent mortality. In the current experiments, for example, both *C. citratus* and *R. officinalis* caused knock down symptoms and a considerable number of moths failed to recover. Therefore, these essential oils should probably not be considered only as repellents, but also as potential insecticides as well. Consequently, such properties should not be ignored when evaluating the bioactivity of essential oils droplets as repellents, since they can reduce pest damage also due to their potential insecticidal activity.

Considering the droplet size, although the main objective of this study was not to rank, or statistically separate drop size volumes, related information was also obtained and may be comparable to other studies. The drop size calibration method, although rather simple compared to other [[60], [61], [62]], provide means to determining and applying the desired droplet spectrum and may help to minimize the spray drift and problems that followed by. Moreover, the frequency of drop size volumes is well described according to the exponential models that were applied, while the effect of pressure on drop size volumes seems to follow a power law. However, in most cases drop diameter falls in the range of 80 μm–400 μm, which usually calls for mist (100 μm) and fine sprays (100–400 μm), having medium to large size droplets, compared to commercial aerosols and fogs. The latest are defined as very fine particles or droplets suspended in air (and ranging in size from 0.1 to 50 μm). At a first look such kind of variations may represent a disadvantage for the reliability of plant treatments in field trials. For vector control with space sprays for instance, the droplet size should be less than 30 μm volume median diameter [59,[63], [64], [65]]. Nevertheless, it is most desirable for Lepidoptera and related fruit pest species, to generate larger droplets (i.e. ∼100 μm) to meet specific application scenarios such as deposing essential oils in which droplets should be retained on host surface for a period of time and form repellent and deterrent plant barriers.

Among the three essential oils tested, *G. procumbens* had weaker oviposition deterrent effects in contrast to *C. citratus* and *R. officinalis* which have affected the number of eggs laid in choice experiments. These findings are in accordance with Paranagame et al. [66] and Papachristos and Stamopoulos [67] who respectively found that *C. citratus* and *R. officinalis* oils were effective against certain insect and that of Setiawati et al. [68], which showed, in choice and non-choice tests, that concentrations of citronella oil (the major component of *C. citratus*) reduced egg laying in *Helicoverpa armigera* by 53–66%. The practical implication of these findings is that when an oviposition substrate is exposed to essential oil drops, satisfactory deterrents may be achieved few days after application even if some insect survive a direct exposure and lay eggs. Behavioral and sub lethal effects have been also demonstrated for major essential oil components such as the structurally related lower terpenes for *Myzus persicae* [69]. However, it should be also noted that the registered adverse effects in egg production may be related to the female’s shorter life spans and not only on indirect effects of essential oils on fecundity and adult maternity.

In most cases oviposition deterrence was higher during early exposure and although the treated substrate was less favorable to females this trend diminished progressively. Therefore, it is possible that the essential oils drops readily impinge on host surface, short time after their application, but the activity weakens as the essential oils concentrations evaporate. This suggestion is supported by the recovery of relatively small number of eggs that were deposited on the oviposition substrate 10 and 15 days after treatment. Nevertheless, other factors may also affect female oviposition preference and may diminish the effectiveness of essential oils, especially under laboratory and field conditions. Fruit varieties for example with rough surfaces [70] or color (Sidney et al.) [71], may also affect oviposition preference. Thomson [72] and Javed et al. [73], stated that the females of *H. armigera* prefer to lay eggs on plant parts with high trichome density and high concentration of stimulatory chemicals. Trichome is a factor which positively affects oviposition preference also in *Anarsia linetaella* [52]. Moreover, fruit damage due to other causes, for example damage by other insect species, is likely to affect fruit protection by essential oils, unless they contain strong deterrent compounds [70]. Nevertheless, since I used the same oviposition substrate in choice experiments, the failure to lay eggs on fruits treated with essential oils was likely due to the high volatile properties and strong essence of the oils, though the presence of non volatile deterrents cannot be eliminated.

Concerning the observed differences among the compounds tested it may be attributed to the specific chemical nature of the essential oils which may possess higher or lower bioactivity properties. Correlating the observed toxicity of essential oils (i.e. rosemary, lavender and eucalyptus) with their chemical composition revealed that their insecticidal properties depend on their total oxygenated monoterpenoid content [74]. Moreover, among the monoterpenoids tested the monocyclic: 1,4-terpineol, limonene, the bicyclic 1,8-cineole and the acyclic linalool, citronellal, displayed strong insecticidal properties to certain species [19].

Thus, the chemical nature of *C. citratus* essential oils may have contributed to its weaker activity since the main constituents are the low toxicity aldehydes citral and neral (70% in content) [75], the monoterpene myrcene and the terpenic alcohol geranial [75]. On the other hand, *R. officinalis*, are reach in high toxicity terpenic alcohols such as 1,8-cineole (35.32%), trans-caryophyllene (14.47%), borneol (9.37%), camphor (8.97%), α-pinene (7.9%) and α-thujone (6.42%). Finally, the fact that the primary constituents of *G. procumbens* oil are very poor in monoterpenoids content [76,77], in which limonene is the most dominant (2.17%), but are very rich in methyl salicylate (96.9%) which possess antimicrobial properties [78], may explain the weak bioactivity that was register in most cases.

Hence, the high bioactivity of the essential oils that were tested could be attributed to some of its major constituents such as citronella, linalool, and 1,8-cineole, monoterpens of high bioactivity [19,79,80]. High toxicity of some these compounds was reported against stored product insects such as the rice weevil *Sitophilus oryzae* and *Rhyzopertha dominica* [81] and *Tribolium confusum,* Stanmopoulos et al [19]. Boeke et al. [82], reported that the volatile oils of *C. nardus* caused most of the eggs not to develop into adult (abnormality in egg development to adult).

Moreover, as has the current study demonstrated, the bioactivity of such substances should be evaluated in the form of ULV fine droplets as well, rather than be administrated solely through their diet, a practice followed by many researchers including agricultural pests [[25], [26], [27], [28], [29]], including tephritids such as *Ceratitis capitata* (Wiedemann) [83], *Bactrocera oleae* [84], *Bactrocera dorsalis* Hendel and *Bactrocera cucurbitae* Coquillet [85], as well as aphids [86] and mosquitos [87].

From a practical point of view, the current laboratory bioassays can be used to evaluate the bioactivity of essential oils and other bio-rational compounds, which exert multilateral influence during a short limited period after their application. However, in contrast to traditional pesticides (i.e. pyrehtroids, some organophosphates and carbamates), in which the insecticidal properties are present at the time of application; essential oils may express their multilateral mode of action few hours and/or days later. Thus, although the use of essential oils in IPM is delicate because of the multiplicity of their phytochemical patterns and bioactivity, in the possible use of essential oils under field conditions it should be borne in mind that longer exposure periods are needed to be affective.

## Conclusion

The device which has been developed can be used to establish olfactory trials under laboratory conditions using Ultra Low Volumes. Moreover, the screening of potential compounds under laboratory condition may save time and cost before filed testing. One important advantage of using ULV formulations of essential and potentially other compounds is to express bioactivity with the lowest concertation. According to the current results all three essential oils that were tested causing repellence and in some cases oviposition deterrence when sprayed in the moth environment in the form of ULV droplets. Moreover, a part of the repellent activity the essential oils may act as insecticides and cause knock down symptoms and mortality. Among the essential oil tested, *R. officinalis* was most active compared to *C. citratus* and *G. procumbens*. Additionally, it is possible that oils may readily impinge on host surface and act also as ovipostion deterrents. Consequently, these properties can be exploitable in two different applications: either as repellents/insecticides and oviposition deterrents. Though the applied method and these findings are limited to lab conditions, they could form a basis for further bioassay improvement and investigation of the questions raised in this work. In particular, additional field research is needed in conjunction with the *in vitro* method trials to improve our understanding insecticidal properties of essential oils and to verify the effect of essential oils on natural populations and under field conditions and this is the object of the next designed study. Finally, the method can be applied to screen the bioactivity of ULV volumes of other compounds as well.
